# Studies on the photodegradation of red, green and blue phosphorescent OLED emitters

**DOI:** 10.3762/bjoc.9.245

**Published:** 2013-10-11

**Authors:** Susanna Schmidbauer, Andreas Hohenleutner, Burkhard König

**Affiliations:** 1 Institut für Organische Chemie, Universität Regensburg, Universitätsstraße 31, D-93053 Regensburg, Germany

**Keywords:** iridium complexes, OLED, photoinduced degradation, phosphorescent emitters

## Abstract

The photodegradation behavior of four well-established iridium emitters was investigated. Irradiation of the samples in different solvents and under atmospheric as well as inert conditions helped to identify several pathways that can contribute to the deterioration of these compounds. Degradation via singlet oxygen or the excited states of the emitters as well as the detrimental influence of halogenated solvents are discussed for the different investigated iridium complexes. Some of the resulting degradation products could be identified by using LC–MS or other analytical techniques. The results show how even small structural changes can have a huge influence on rate and mechanism of the photodegradation. The observations from this study may help to better understand degradation processes occurring during the handling of the materials, but also during device processing and operation.

## Introduction

For applications such as displays or lighting, organic light-emitting devices have to enable the efficient conversion of electrons into photons to ensure sufficient light output at low power consumptions. To achieve this, the use of organotransition metal complexes as phosphorescent dopants has proved highly beneficial. While fluorescent dyes normally emit from their singlet excited states (at room temperature) and therefore can only utilize singlet excitons, phosphorescent organotransition metal dopants emit from their triplet excited states. In addition phosphorescent organotransition metal dopants are able to effectively convert singlet into triplet states via fast intersystem crossing. The recombination of electron–hole pairs generates singlet and triplet excitons in a ratio of 1:3 which means that the use of phosphorescent devices can give up to 4 times higher efficiencies than those based on fluorescent emitters – which is called the triplet harvesting effect [1]. However, while OLEDs with efficiencies comparable to fluorescent tube lamps have been realized [2], the operational stability of the devices remains to be a challenge in this field. In particular, the chemical degradation of the materials during operation is still considered the major obstacle for the development of economically feasible devices. In many cases of highly efficient phosphorescent devices it is indeed likely that the luminance loss over time can be in part or even largely attributed to the deterioration of the emitters [3–4]. The development of highly stable phosphorescent dopants is therefore a topic of high interest. Thus, investigating the mechanisms and pathways responsible for the degradation of these materials is of high importance as the insights from these studies can guide the development of new materials with enhanced stabilities. Most studies on the degradation mechanisms of materials in OLEDs in general and of the phosphorescent emitters in particular suggest the participation of excited states [3,5–13]. This can proceed via direct instability of the excited states, or via higher lying unstable states that are accessible via annihilation reactions [14–15] or thermal population [16]. Investigations on the photodegradation of these compounds could therefore potentially provide valuable information on the processes that are responsible for the limited stability of phosphorescent OLEDs.

In a previous study we already used the photoinduced degradation of phosphorescent iridium complexes in solution as a fast screening tool for their degradation behavior and observed interesting differences and effects in correlation to the examined structures [17]. In this context, we wanted to gain a deeper understanding of the processes at work and to examine whether the photostability in solution and in a solid organic matrix would show a similar behavior. We therefore selected four well-known phosphorescent emitters for a detailed investigation of their photodegradation behavior in solution and in thin polymer films. To gain information on the chemical degradation mechanisms, we tried to identify deterioration products by liquid chromatography mass spectrometry (LC–MS) and other analytical methods where applicable.

## Results and Discussion

**Figure 1:**
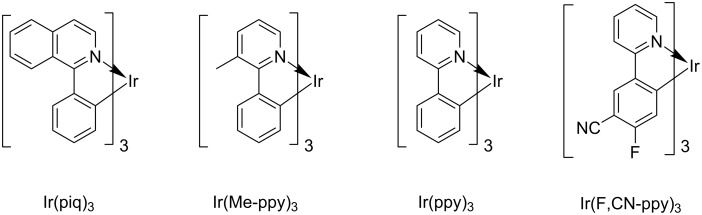
Structures of the investigated phosphorescent triscyclometalated iridium complexes.

For our investigations, we chose the four iridium complexes depicted in Figure 1. The compounds exhibit emission in the red (Ir(piq)_3_), green (Ir(Me-ppy)_3_ and Ir(ppy)_3_) and blue region (Ir(F,CN-ppy)_3_), respectively. Especially Ir(piq)_3_ and Ir(ppy)_3_ are popular phosphorescent emitters and often used in devices. For the investigation of the behavior of these compounds under continuous excitation, the samples were irradiated with light of 400 nm high power LEDs (350 mW). This allows for the excitation of the MLCT absorption band and further enables the irradiation through standard chromatography glass vials and glass substrates without having to resort to quartz cuvettes. Toluene was chosen as a solvent since it is a comparably inert non-coordinating solvent that is also preferably used for the processing of OLEDs from solution. To check for an influence of the solvent, we also performed experiments in CH_2_Cl_2_. To be able to compare the behavior in solution and solid state, we further prepared thin poly(methyl metacrylate) (PMMA) films doped with the different materials by spin coating and subjected them to irradiation under the same conditions. For the degradation studies in solution, the ratio of remaining to initial complex concentration after the different irradiation times was determined by HPLC analysis, for the solid samples by measuring the luminescence intensities of the samples. The majority of the experiments was performed at least twice and was reproducible within an estimated error of <10%. The degradation curves were generated by using the average value of the obtained data (the results of the individual experiments are provided in Supporting Information File 1). We will first discuss general trends and observations and then elaborate on the role of the halogenated solvents followed by a more detailed discussion for each of the investigated behavior of the compounds.

### General observations

**Figure 2:**
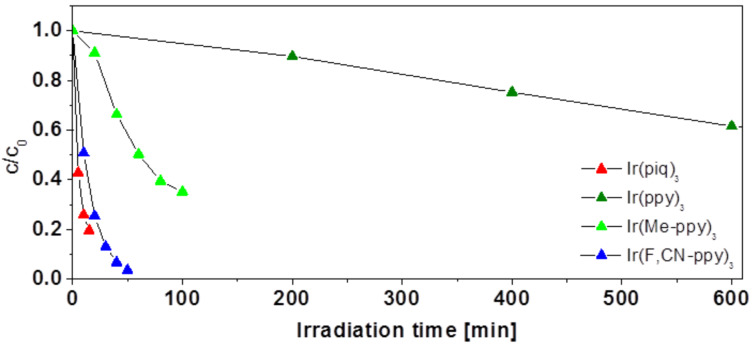
Photodegradation of the different compounds in toluene under ambient atmosphere. Plotted is the ratio between the remaining and initial concentrations for the respective compound against the irradiation time.

The rate of photodegradation shows substantial differences as can be seen in Figure 2, which compares the concentration decrease of the four substances in toluene under ambient conditions. While Ir(ppy)_3_ is fairly stable, Ir(Me-ppy)_3_ is degrading considerably faster and Ir(piq)_3_ as well as Ir(F,CN ppy)_3_ exhibit an extremely fast deterioration in the course of only a few minutes. While this general trend for the stability of the different complexes (Ir(ppy)_3_ >> Ir(Me-ppy)_3_ > Ir(piq)_3_/Ir(F,CN-ppy)_3_) was found to apply to all examined conditions, the matrix can have a pronounced influence on the degradation rate.

**Figure 3:**
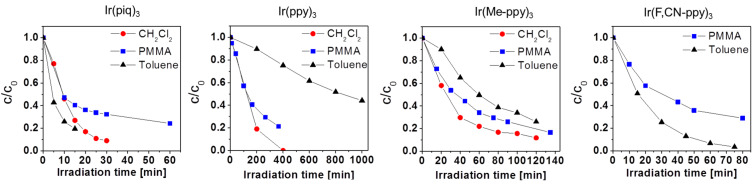
Photodegradation behavior of the iridium complexes in CH_2_Cl_2_ and toluene solution and in a spin-coated PMMA polymer film under ambient conditions. For measurements that were performed repeatedly, the depicted values represent the mean of all experiments.

All compounds exhibit at least equal or higher stabilities in toluene compared to CH_2_Cl_2_ (no reproducible results could be obtained for Ir(F,CN-ppy)_3_ in CH_2_Cl_2_). Interestingly, deterioration in the solid PMMA matrix also follows the overall trends as observed for the experiments in solution. This may be an indicator that the deterioration of these compounds might be indeed based on – at least partly – the same processes in solution and solid state. Figure 3 shows the degradation in different matrices at ambient conditions for all four compounds (note the different scale for the separate graphs).

### The influence of halogenated solvents

**Figure 4:**
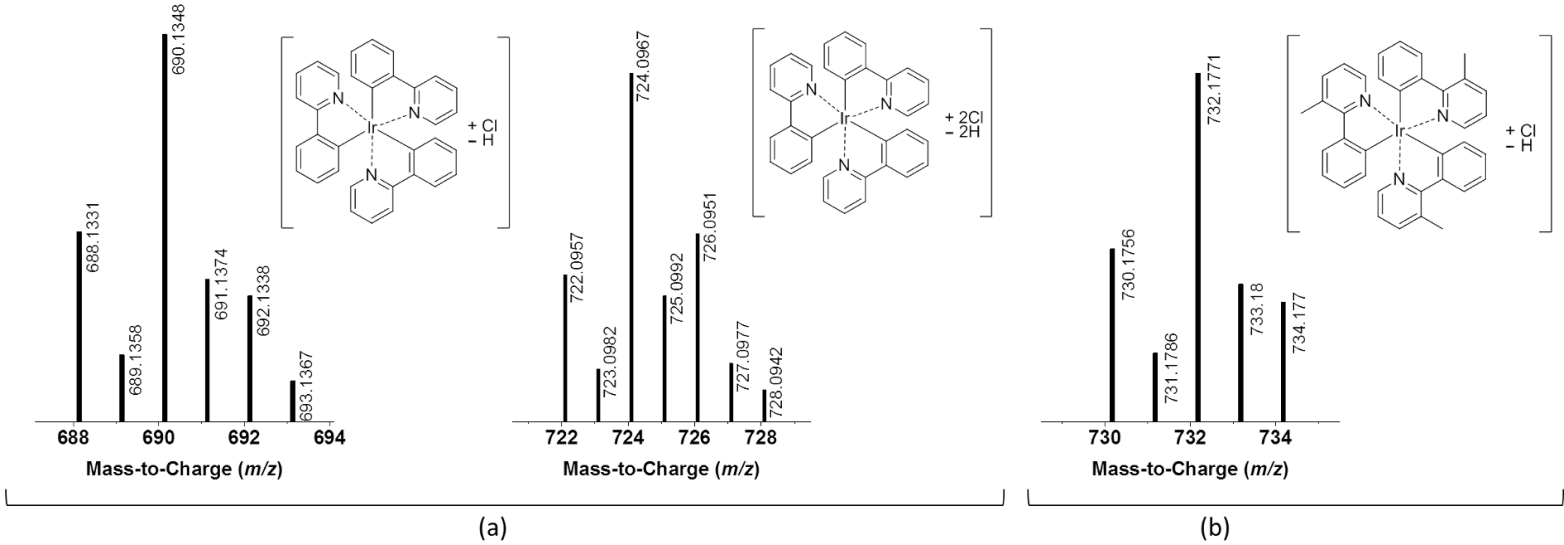
Mass spectra of the identified halogenated degradation products of a) Ir(ppy)_3_ after 200 min of irradiation in CH_2_Cl_2_ and b) Ir(Me-ppy)_3_ after 120 min of irradiation in CH_2_Cl_2_ (obtained from the LC–MS analysis of the respective samples).

Though halogenated solvents often provide the best solubility for this class of compounds, it is generally believed that their use is detrimental to the stability of these materials. The observed higher degradation rates of the compounds in CH_2_Cl_2_ seem to support this assumption. The higher the general stability of the investigated compounds, the stronger the detrimental effect of the use of CH_2_Cl_2_ seemed to be. While there is little to no influence of the solvent observable for Ir(F,CN-ppy)_3_ and Ir(piq)_3_, it is more pronounced for Ir(Me-ppy)_3_ and becomes particularly striking for Ir(ppy)_3_ with the degradation being about eight times faster in CH_2_Cl_2_ compared to toluene. A possible explanation for this observation could be that solvent independent processes are responsible for the generally very low photostabilities of the red and blue emitter and the solvent effect is therefore negligible. With the overall higher stabilities of compounds that emit green light however, processes associated with CH_2_Cl_2_ as the solvent become apparent. LC–MS analysis indeed revealed that the main observed degradation products of Ir(ppy)_3_ after irradiation in CH_2_Cl_2_ were chlorinated species, formed by the exchange of one or two hydrogen atoms for a chlorine substituent (Figure 4). This can be inferred from the *m*/*z* values as well as isotope distribution patterns. Analogous products could also be observed for Ir(Me-ppy)_3_, though only as a side product, while no indication of halogen abstraction was found for the other two complexes.

### The influence of dioxygen

As cyclometalated Ir-complexes are very efficient singlet oxygen sensitizers we investigated the influence of oxygen on the photostability of the compounds. ^1^O_2_ is known to undergo a variety of reactions with nitrogen heterocycles [18] and might thus attack the emitters, leading to the formation of a variety of deterioration products. We therefore performed the irradiation experiments in different solvents under atmospheric as well as inert conditions (freeze-pump-thaw, then argon atmosphere). All compounds were irradiated in CH_2_Cl_2_ and toluene as mentioned above, but also in benzene and benzene-*d*_6_. Singlet oxygen has a significantly higher lifetime in deuterated solvents, resulting in an effective enhancement of the reaction rates of singlet oxygen. Processes induced by this highly reactive species should therefore be significantly faster in these solvents. The comparison of degradation rates in benzene and benzene-*d*_6_ should therefore help to elucidate the role singlet oxygen plays in the degradation of these materials as the degradation should be faster under atmospheric conditions than under inert atmosphere and show a further increase when performed in benzene-*d*_6_. Iridium complexes have been proposed to not only undergo energy transfer to dioxygen but also being capable of electron transfer leading to the formation of superoxide anions. Especially for the blue emitting Ir(F,CN-ppy)_3_ with its higher emission energy, a participation of this mechanism is likely. However, it was shown that iridium complexes exhibit singlet oxygen quantum efficiencies close to the photoluminescence quantum efficiencies of these compounds. Djurovich et al. suggested that back-electron transfer from the superoxide anion to the oxidized sensitizers eventually also leads to the formation of singlet oxygen [19–20]. It is noteworthy that when we are talking about degradation processes by singlet oxygen, a participation of superoxide anions cannot be completely ruled out.

Other processes, possibly competing with the above mentioned reactive oxygen species-induced degradation route may proceed via the excited state of the emitter molecule. This might be due to instability of the excited molecule itself, via interaction of the excited molecule with its local environment or even other excited states [14,16,21]. Supposed the molecule is not susceptible to an attack of ^1^O_2_, it would be expected that the presence of oxygen in this case actually increases the photostability of the material. This is due to the very fast quenching of the excited state by O_2_ [22], leading to a significantly shorter lifetime of the excited states and consequently a reduced degradation rate for processes induced via those states. The observations from the photodegradation experiments suggest strong differences in the contribution of these two pathways for the four examined compounds. We therefore discuss the photodegradation behavior and the observed degradation products individually for each of the emitters.

The investigations in CH_2_Cl_2_ under both ambient and inert conditions showed the same trend as those in benzene for all complexes. Within the range of measurement accuracy, no differences in the degradation behavior between experiments in toluene and benzene were observable. For clarity, only the results of the measurements in benzene and benzene-*d*_6_ are therefore depicted and discussed in the following part (Supporting Information File 1 provides the additional experimental degradation data).

### Ir(piq)_3_

**Figure 5:**
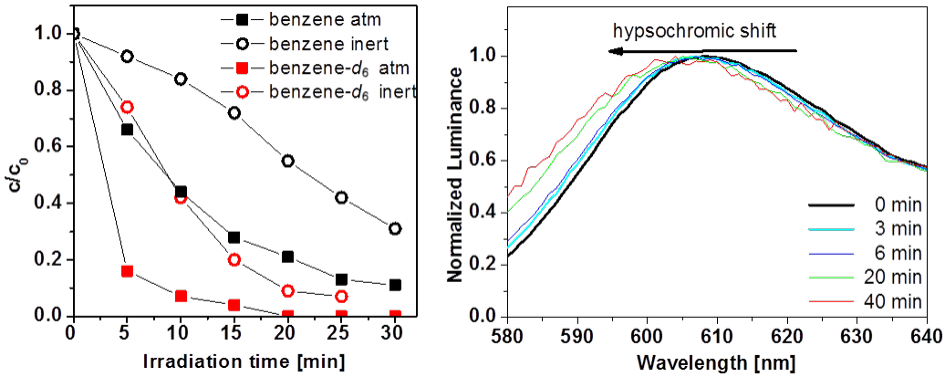
Degradation curves of Ir(piq)_3_ in benzene and benzene-*d*_6_ under ambient and inert conditions (left). Hypsochromic shift of the phosphorescence of an Ir(piq)_3_-doped PMMA film with increased irradiation time (right).

As mentioned above, red emitting Ir(piq)_3_ is one of the most unstable compounds in the series of complexes investigated here. Irradiation in benzene under atmospheric conditions led to a fast degradation with only about 10% of the initial concentration after 30 min of irradiation (Figure 5). Under the exclusion of oxygen, however, the degradation is suppressed significantly. Therefore, reactive oxygen species are likely responsible for the decomposition of Ir(piq)_3_. Experiments in deuterated benzene under atmospheric conditions support this assumption showing more than 80% decrease in the complex concentration after only 5 min of irradiation. The observation that the degradation is also considerably faster in deuterated benzene under inert conditions might be attributed to the fact that a quantitative removal of oxygen is unlikely by the employed procedure. The repeated punctuation of the septa by the injector needle of the HPLC-autosampler might further allow small quantities of oxygen to enter the sample vial. This suggests that only traces of oxygen can have a pronounced effect on the durability of the complexes. Incorporation of the complex in a PMMA matrix leads to similar degradation rates at the beginning of the experiment (Figure 3). However, after 15–20 min of irradiation, the luminance changes decrease significantly. A possible explanation is the formation of degradation products which are still capable of phosphorescent emission, albeit at lower luminescence quantum efficiencies. This rational is supported by comparing the normalized phosphorescence spectra after consecutive irradiation periods (Figure 5). A hypsochromic shift and signal broadening with increased irradiation times is observed, indicating the presence of other species contributing to the emission.

**Figure 6:**
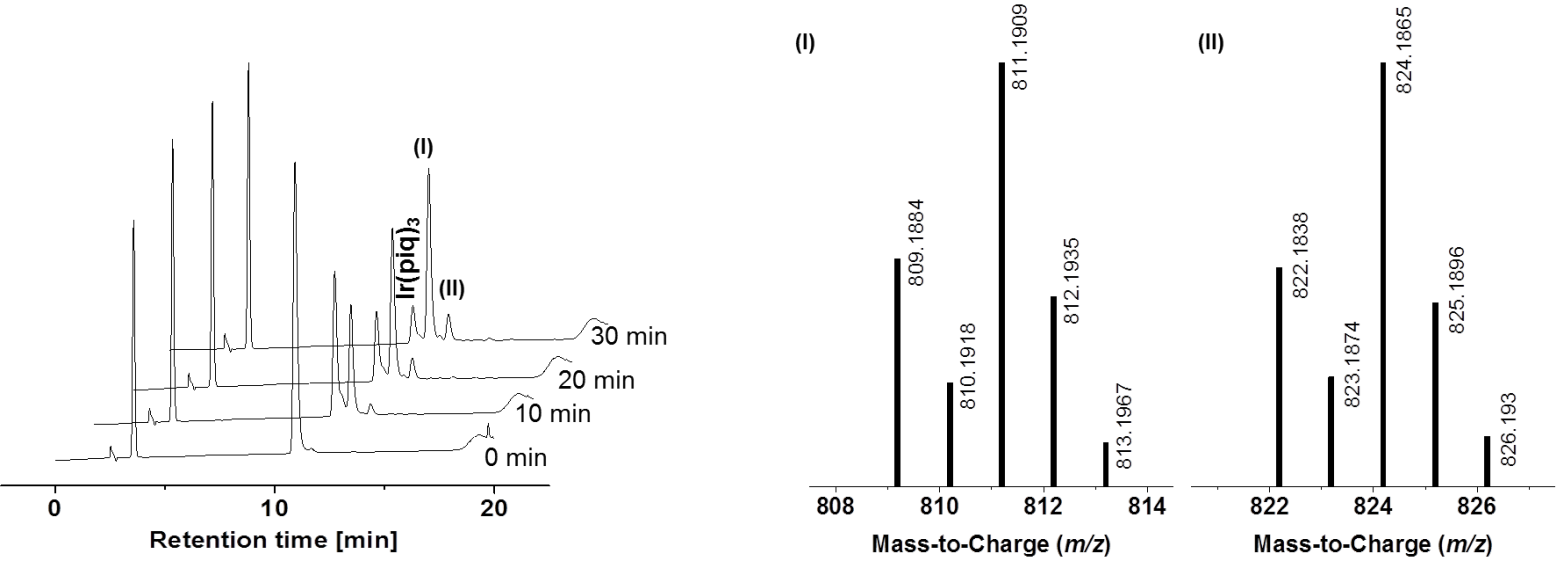
DAD chromatogram showing the formation of two main degradation products (left) and their assigned mass spectra (right).

In fact, the HPLC–DAD chromatograms of the soluble samples show that the decrease of the complex signal is accompanied by the emergence of two new peaks (Figure 6). These degradation products can be assigned to the *m/z* values 811 (I) and 824 (II) respectively. Despite NMR investigations of the decomposed sample, the structure of these two products could not be elucidated so far.

### Ir(ppy)_3_

**Figure 7:**
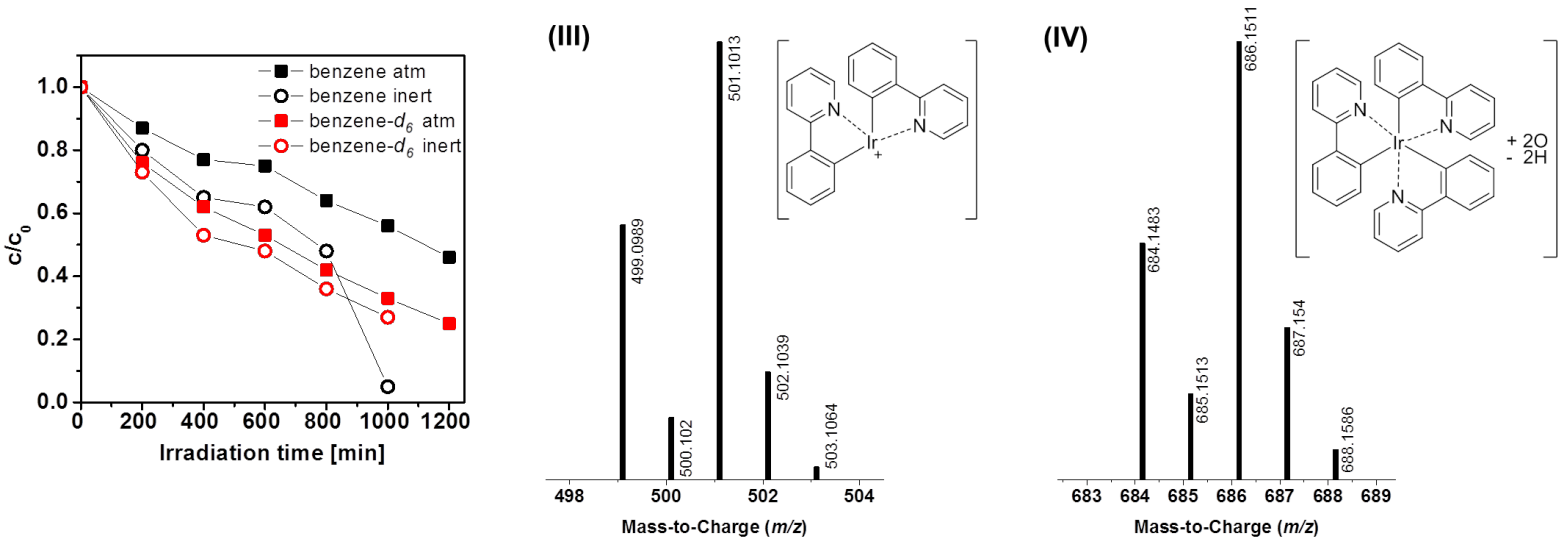
Degradation curves of Ir(ppy)_3_ in benzene and benzene-*d*_6_ under ambient and inert conditions, and mass spectra of two degradation products with the assigned structures.

In contrast, for the green emitting Ir(ppy)_3_ the presence of oxygen in benzene has a beneficial effect on the compound’s stability. This is likely due to the ability of oxygen to quench the triplet excited state of the complex, thus preventing degradation through this state (Figure 7). Newly formed species, substantiating the contribution of the excited state on the complexes degradation, were identified via HPLC–MS analysis: the *m*/*z* value of 501 can be assigned to a species formed by the dissociation of one phenylpyridine ligand (III). This literature-known phenomenon can be explained by the population of higher lying metal-centered states leading to bond rupture [7,9–10,23]. Mass spectrometric investigations revealed the formation of three additional degradation products with the *m*/*z* values 603, 632 and 686, respectively. For the latter, an oxygenated species [Ir(ppy)_3_ + 2O − 2H] (IV) can be assumed. However, the very small amounts did not allow for additional analyses by NMR to confirm the suggested structures.

### Ir(Me-ppy)_3_

**Figure 8:**
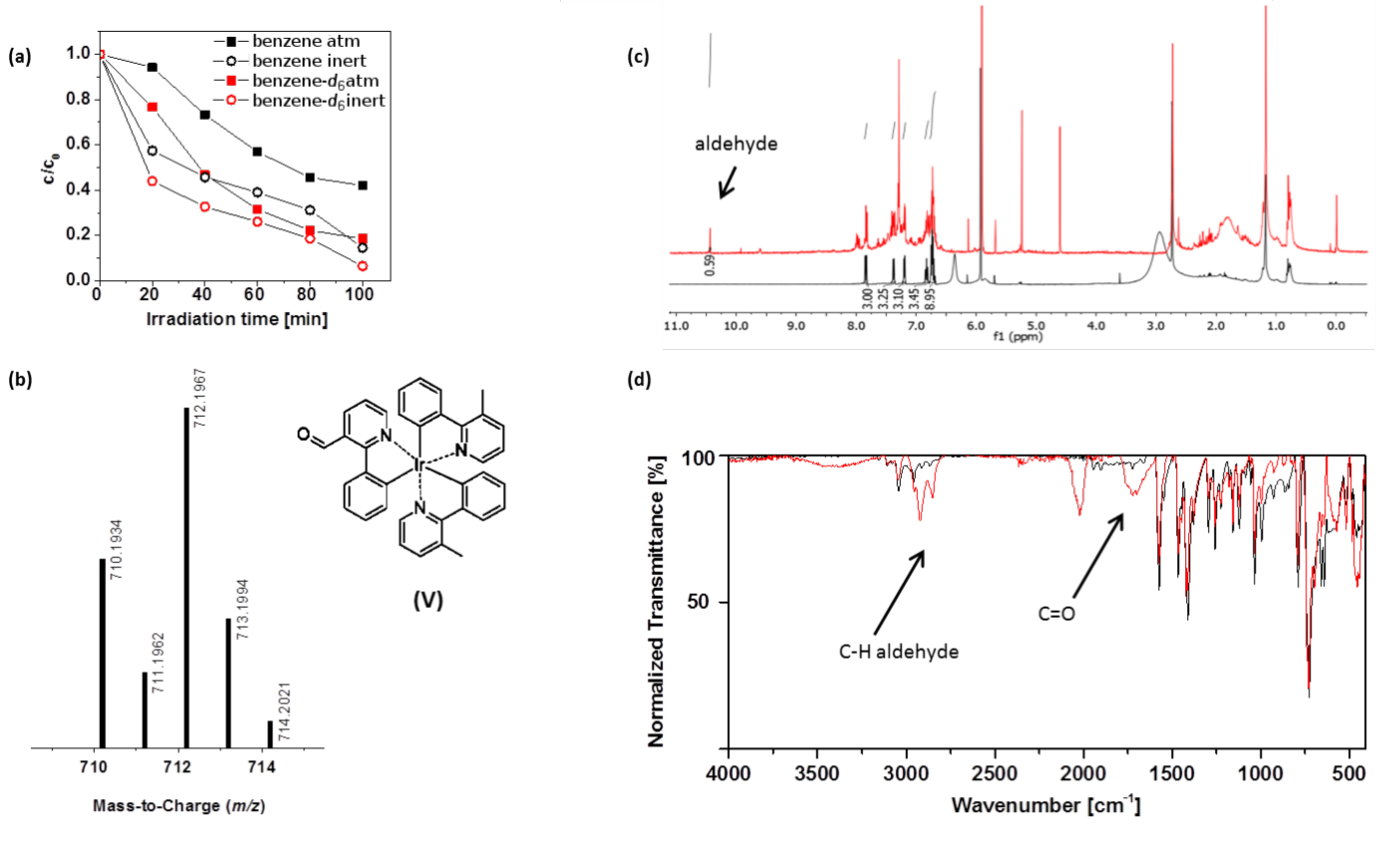
(a) Degradation curves of Ir(Me-ppy)_3_ in benzene and benzene-*d*_6_ under ambient and inert conditions. (b) Mass spectrum of the main degradation product with its assigned structure. The aldehyde functionality can be visualized by NMR (c) and IR (d) spectroscopy (black: non-irradiated sample, red: irradiated sample).

As already observed for Ir(ppy)_3_, the methylated derivative Ir(Me-ppy)_3_ degrades much faster under inert than under atmospheric conditions in benzene (Figure 8a), which indicates the degradation by excited-state-induced processes. The same trend can be observed in benzene-*d*_6_ albeit with a significantly diminished stability. According to the DAD chromatogram, the decrease of the complex signal with the irradiation time was accompanied by the formation of only one visible degradation product with a mass of 712 (MH^+^ + 14). This species was attributed to the aldehyde functionalized complex derivative (V, Figure 8b), as the carbonyl functionality was confirmed by NMR spectroscopy with a signal at 10.5 ppm as well as IR spectroscopy with a typical signal at around 1700 cm^−1^ for the C=O stretching frequency and two signals at around 2800 cm^−1^ for the C–H stretching frequencies (Figure 8c and Figure 8d). As it is known that singlet oxygen can oxidize methyl-substituted aromatic systems to form aromatic aldehydes [24], this proposed structure is in good agreement with the course of the degradation curve of the experiments in benzene-*d*_6_ under ambient conditions. The increased lifetime of ^1^O_2_ in the deuterated solvent enhances the complex oxidation. Nonetheless, the observation that the degradation proceeds is fastest under inert conditions in deuterated benzene cannot be explained with this deterioration model. As this behavior can also be observed for the green analogue Ir(ppy)_3_ (Figure 7) the contribution of another, still unidentified deterioration pathway is most likely.

### Ir(F,CN-ppy)_3_

**Figure 9:**
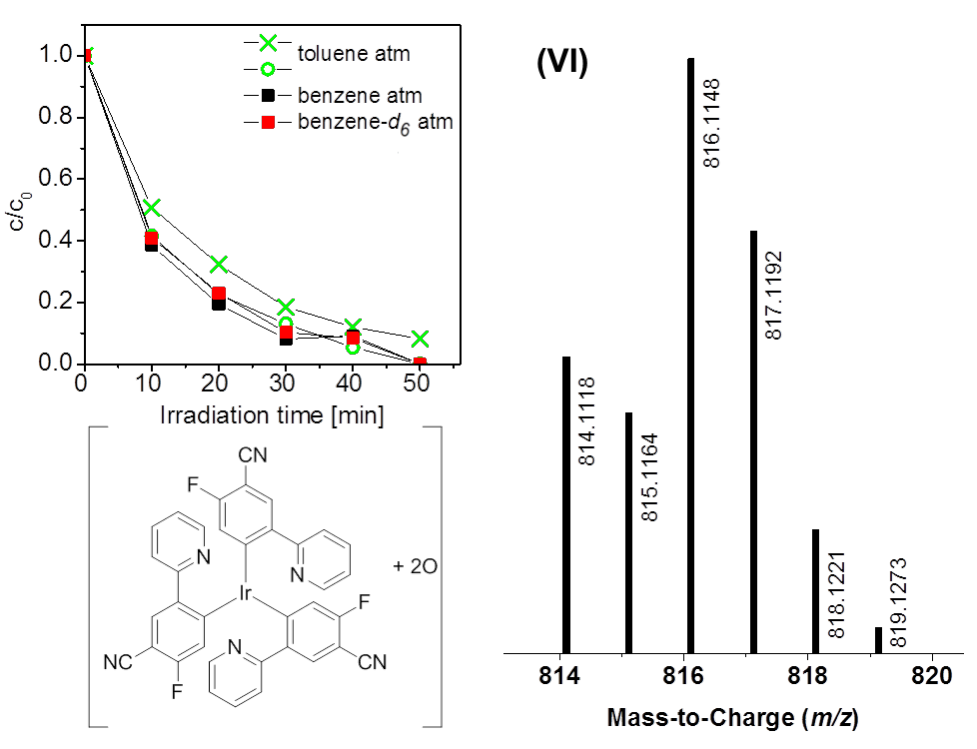
Degradation curves of Ir(F,CN-ppy)_3_ in benzene, benzene-*d*_6_ and toluene under ambient conditions and mass spectrum of the detected degradation product, which matches a twofold-oxidized derivative with regard to its *m*/*z* value and the corresponding isotope pattern.

The blue emitting Ir(F,CN-ppy)_3_ brought some difficulties in the elucidation of its degradation behavior. The solubility of this compound in all chosen solvents was poor and the complex turned out to be very unstable in solution even without irradiation: after one day in the dark, the DAD signal of this complex decreased by half in toluene, benzene and benzene-*d*_6_. Therefore, the reproducibility of the experiments was not satisfying. Reliable results were only achieved for the measurements in toluene under atmospheric conditions, with an estimated error of approximately 10% (Figure 9). The experiment in benzene and benzene-*d*_6_ under ambient conditions showed a similar degradation behavior. This is in good agreement with observations of the solvent dependency for the other emitters. Under inert conditions, no reliable dataset could be obtained. Expected degradation products like species formed by fluorine cleavage, which were reported previously for blue emitting iridium analogues [25–26], could not be observed. This might be due to the generally poor ionization the complex exhibits. Only small traces of one degradation product with the *m*/*z* value 816 were detected (VI), which could be attributed to an double oxidized derivative [Ir(F,CN − ppy)_3_ + 2O].

## Conclusion

In conclusion, we have investigated the degradation behavior of four well-established iridium emitters. The general stability of these complexes in solution and thin solid films follows the trend Ir(ppy)_3_ > Ir(Me-ppy)_3_ > Ir(piq)_3_ (~ Ir(F,CN-ppy)_3_). Irradiation of the samples in different solvents and under atmospheric as well as inert conditions allowed us to suggest several pathways that can contribute to the deterioration of these compounds.

CH_2_Cl_2_ as a representative of halogenated solvents has a detrimental influence on the stability of the compounds. The fast deterioration in this solvent and the identification of chlorinated species after the irradiation experiments for Ir(ppy)_3_ and Ir(Me-ppy)_3_ support the general notion that halogenated solvents should be avoided for these complexes when possible.

Utilizing the enhanced lifetime of ^1^O_2_ in deuterated solvents, we showed that the emitters can be very susceptible to singlet oxygen-induced degradation depending on their structure. This pathway was identified as the predominant degradation mechanism for the red emitting Ir(piq)_3_. Ir(Me-ppy)_3_ also showed sensitivity towards reactive oxygen species and an oxidized deterioration product could be identified after irradiation. The very fast degradation under the influence of oxygen and light emphasizes the necessity to not only process the devices, but ideally also handle and store the materials under an inert atmosphere and the exclusion of light whenever possible.

While the mechanisms for these reactions can be manifold and the exact structural identification of specific products is very difficult, the results suggest a strong contribution of degradation via the excited state. These processes are likely also the ones that are most relevant for the operational degradation of these compounds in OLEDs. We could for example observe the dissociation of one phenylpyridine ligand for Ir(ppy)_3_, a product that has been identified in aged OLEDs as well [7].

However, the photodegradation of these materials is complex and likely caused not only by one but many different mechanisms. Observations indicating the contribution of the excited states, singlet oxygen and possibly other unidentified pathways to the degradation of Ir(Me-ppy)_3_ show that the different mechanisms are competing for this emitter.

The results from this study furthermore clearly show how even small changes in the ligand structure can have a huge impact not only on the rate but also on the mechanisms of their degradation. For a proper rationalization of all the above mentioned findings, however, the degradation behavior of many more emitters would have to be studied. The particular striking differences in the degradation of Ir(ppy)_3_ and Ir(Me-ppy)_3_, which only differ in a methyl group, emphasize how difficult it is at this state of research to reliably predict the influence of structural changes on the stability of the emitters. More profound knowledge on the mechanisms and products may contribute to an understanding of the degradation processes occurring during device operation but also to avoid adverse conditions such as halogenated solvents, light or oxygen during preparation, storage and handling of these materials. This can prevent the formation of degradation products that might be detrimental to the stability of organic light-emitting devices.

## Experimental

The complexes were provided by Merck KGaA. The solvents were purchased from Merck KGaA, Acros and Sigma–Aldrich and were used as received without further purification. Toluene and benzene were used in p.a. quality. CH_2_Cl_2_, THF and hexane were LiChrosolv solvents.

### Photodegradation studies of the liquid samples

In a similar manner to a procedure described previously [17], the complexes were dissolved in the appropriate solvent (50 µM) and 1 mL of the resulting solution was transferred to a headspace vial and sealed with a silicone/PTFE septum. Two samples were prepared from each complex for the testing under atmospheric and inert conditions. In case of the photodegradation studies under inert conditions, the vials were degassed via three consecutive freeze-pump-thaw cycles under argon. All samples were irradiated in a custom made irradiation unit (SIM GmbH). It consists of an aluminum-printed circuit board with thirty 400 nm LEDs (350 mW, Edison Edixeon 3 W Emitter, [LT-1467]), connected to a cooling unit, that ensures a constant temperature of 20 °C of the board during the irradiation. Two 15 sample chromatography trays can be placed in the unit so that each of the sample vials is centered over one LED (d = 1 cm). The irradiation intervals were chosen individually for each complex according to the overall lifetime. After each irradiation cycle, the samples were analyzed by HPLC (see below). The remaining emitter concentration was determined by integration of the DAD signal at 305 nm in relation to that of an external standard (quaterphenyl in toluene, 0.026 mM).

#### Analysis of the liquid samples and identification of degradation products

The analysis was performed on an Agilent Technologies 1200 HPLC–MS system consisting of a binary pump SL [G1312B], a degasser [G1379B], an Infinity high performance micro autosampler [G1329B] with thermostat [G1330B], a thermostatted column compartment [G1316B], an Infinity diode-array detector (DAD) [G4212B] and an accurate mass Q-TOF/MS [G6530A] with an APCI (atmospheric pressure chemical ionization) ion source. The column used was a HIbar® 250 mm × 4 mm × 5 µm diol column (Merck KGaA). For NMR and IR investigations, the solvent (toluene) was removed from the irradiated sample in a nitrogen stream. NMR spectra in deuterated tetrachloroethane (TCE-*d*_2_) were recorded on a Bruker Avance 400 (400.13 MHz for ^1^H and 100.03 MHz for ^13^C) spectrometer. IR spectra of the solid samples were recorded on a BIO-RAD Excalibur IR spectrometer.

#### Photodegradation studies of the PMMA substrates

PMMA and the complex (0.17 wt %) were dissolved in CH_2_Cl_2_ and spin-coated on a glass substrate. The films were irradiated as described for the liquid samples. The analysis of the PMMA substrates was performed on a HORIBA Scientific Fluoromax-4 spectrofluorometer by using a solid sample holder, which ensures an accurate positioning of the substrate for the measurement. The *c*/*c*_0_-values were determined via the ratio of the integrated phosphorescence spectra after irradiation to its initial value.

## Supporting Information

File 1 Reproducibility of degradation studies and degradation curves.
