# Supplementary material for: Studies on the photodegradation of red, green and blue phosphorescent OLED emitters
Source: Beilstein J Org Chem. 2013 Oct 11;9:2088–96. doi: 10.3762/bjoc.9.245 (PMC3817512; doi:10.3762/bjoc.9.245)

Supporting Information
for
Studies on the photodegradation of red, green and blue phosphorescent OLED emitters

Susanna Schmidbauer§, Andreas Hohenleutner§ and Burkhard König*

Address: Institut für Organische Chemie, Universität Regensburg, Universitätsstraße 31, D-93053 Regensburg, Germany

E-mail: Burkhard König - burkhard.koenig@chemie.uni-regensburg.de

§These authors contributed equally

*Corresponding author

Reproducibility of degradation studies and degradation curves

Contents

Reproducibility of the degradation studies in toluene under ambient conditions S2

Reproducibility of the degradation studies in a PMMA matrix under ambient conditions S3

Degradation curves of Ir(piq)$_3$, Ir(ppy)$_3$, Ir(Me-ppy)$_3$ and Ir(F,CN-ppy)$_3$ in CH_2Cl_2 under ambient and inert conditions S4

Degradation curves of Ir(piq)$_3$, Ir(ppy)$_3$, Ir(Me-ppy)$_3$ and Ir(F,CN-ppy)$_3$ in benzene and toluene under ambient and inert conditions S5

Reproducibility of the degradation studies in toluene under ambient conditions

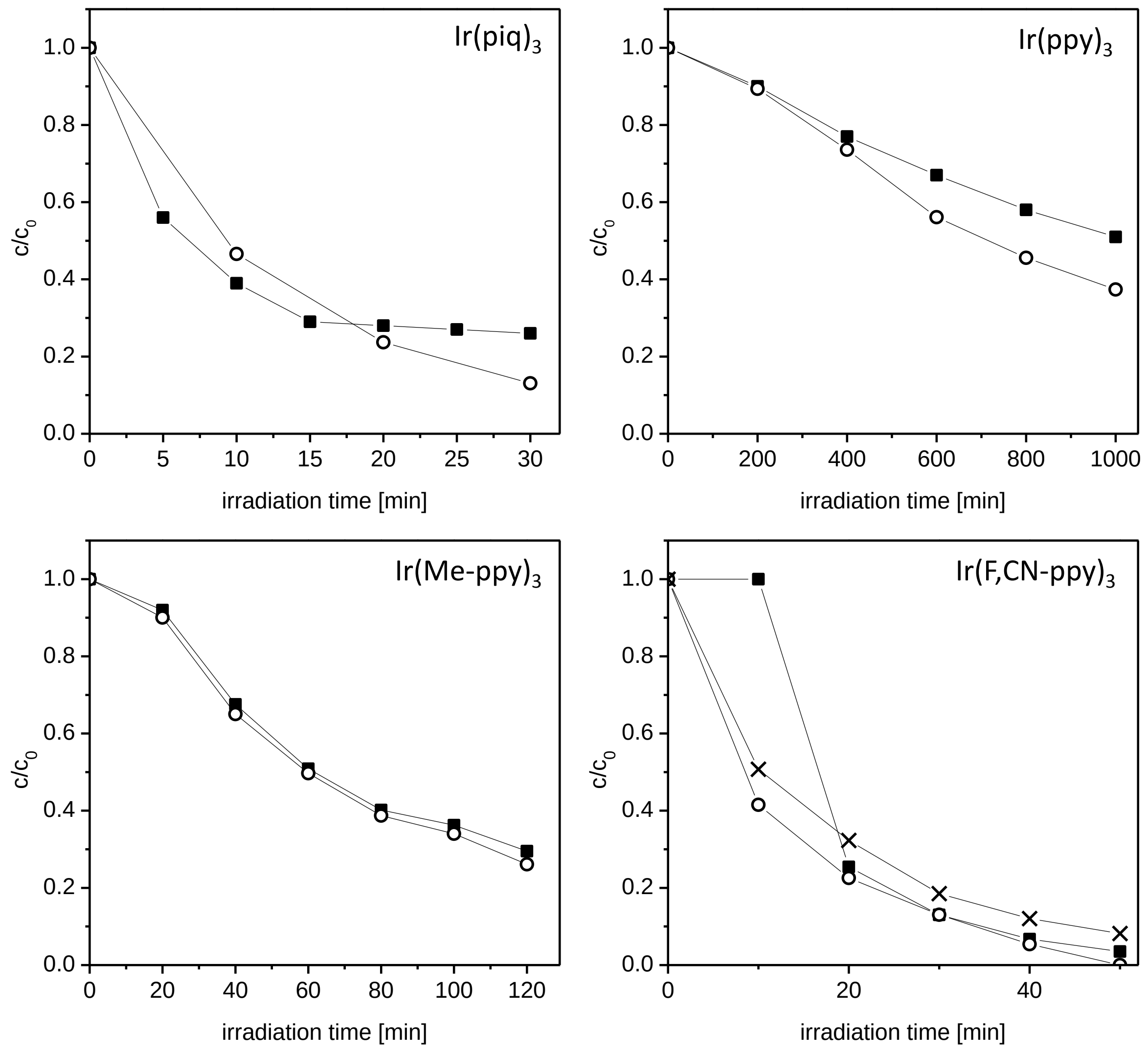

<u>Reproducibility of the degradation studies in a PMMA matrix under ambient conditions</u>

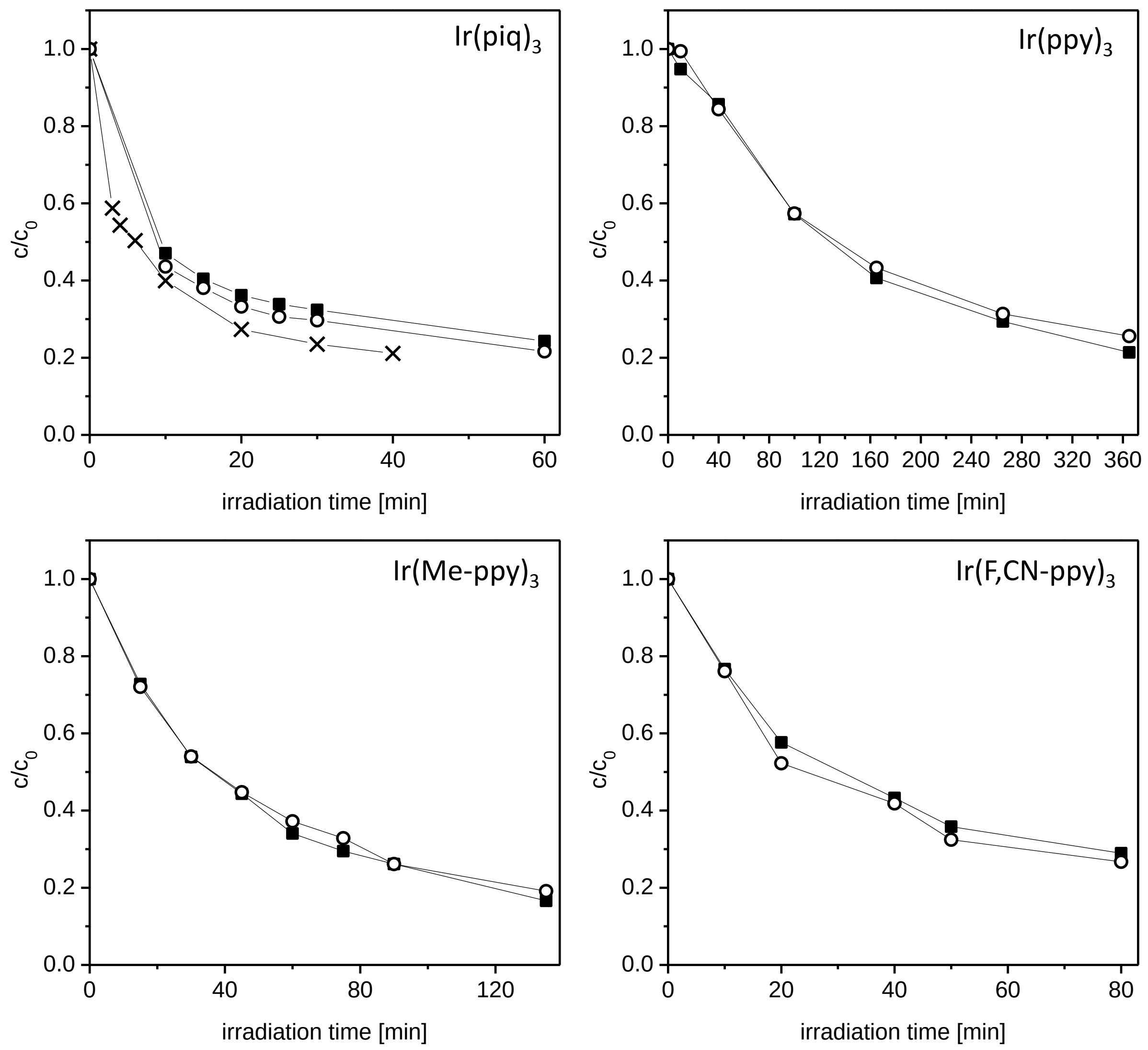

Degradation curves of $Ir(piq)_3$, $Ir(ppy)_3$, $Ir(Me\text{-}ppy)_3$ and $Ir(F,CN\text{-}ppy)_3$ in CH_2Cl_2 under ambient and inert conditions

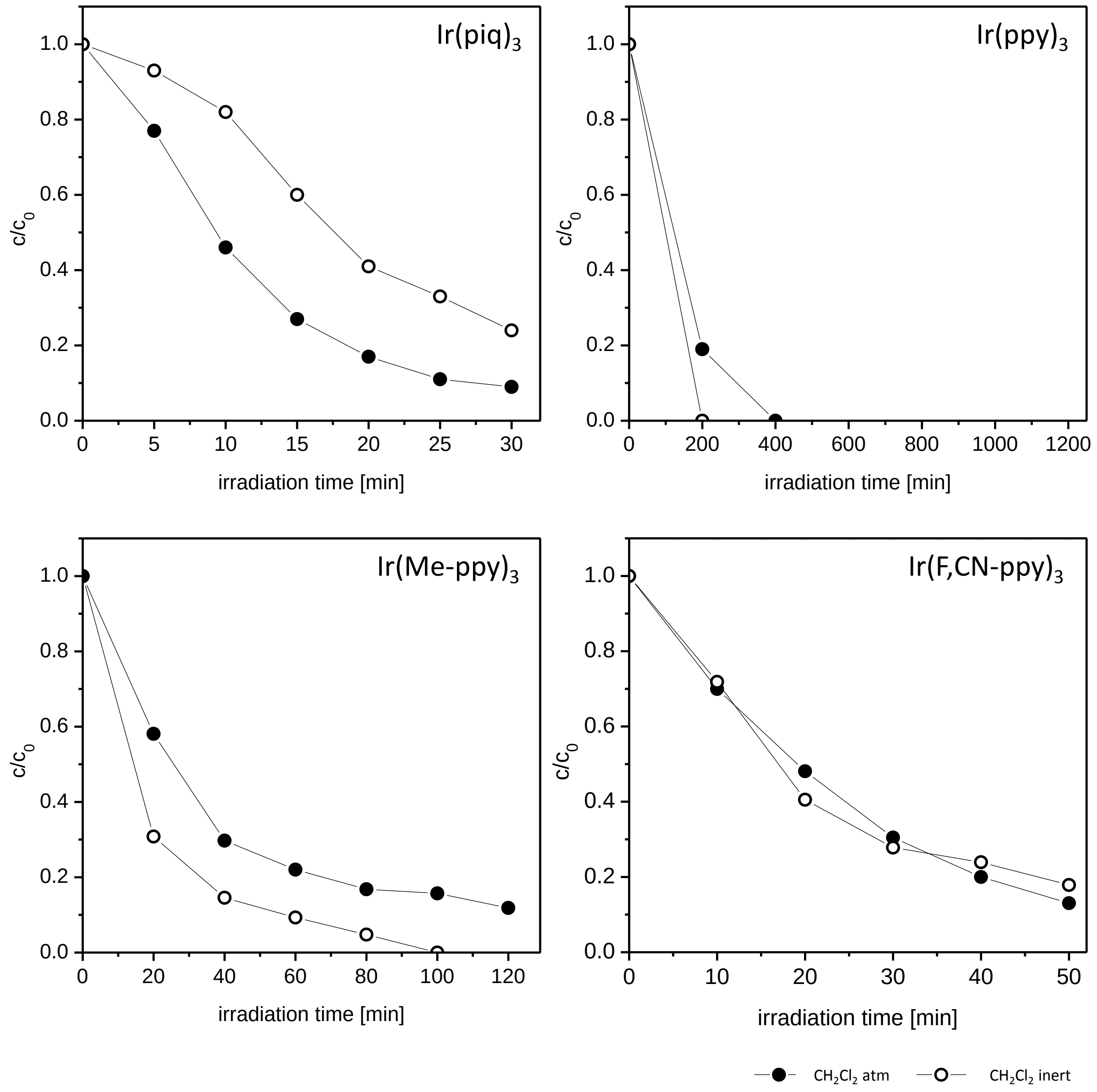

Degradation curves of $Ir(piq)_3$, $Ir(ppy)_3$, $Ir(Me\text{-}ppy)_3$ and $Ir(F,CN\text{-}ppy)_3$ in benzene and toluene under ambient and inert conditions

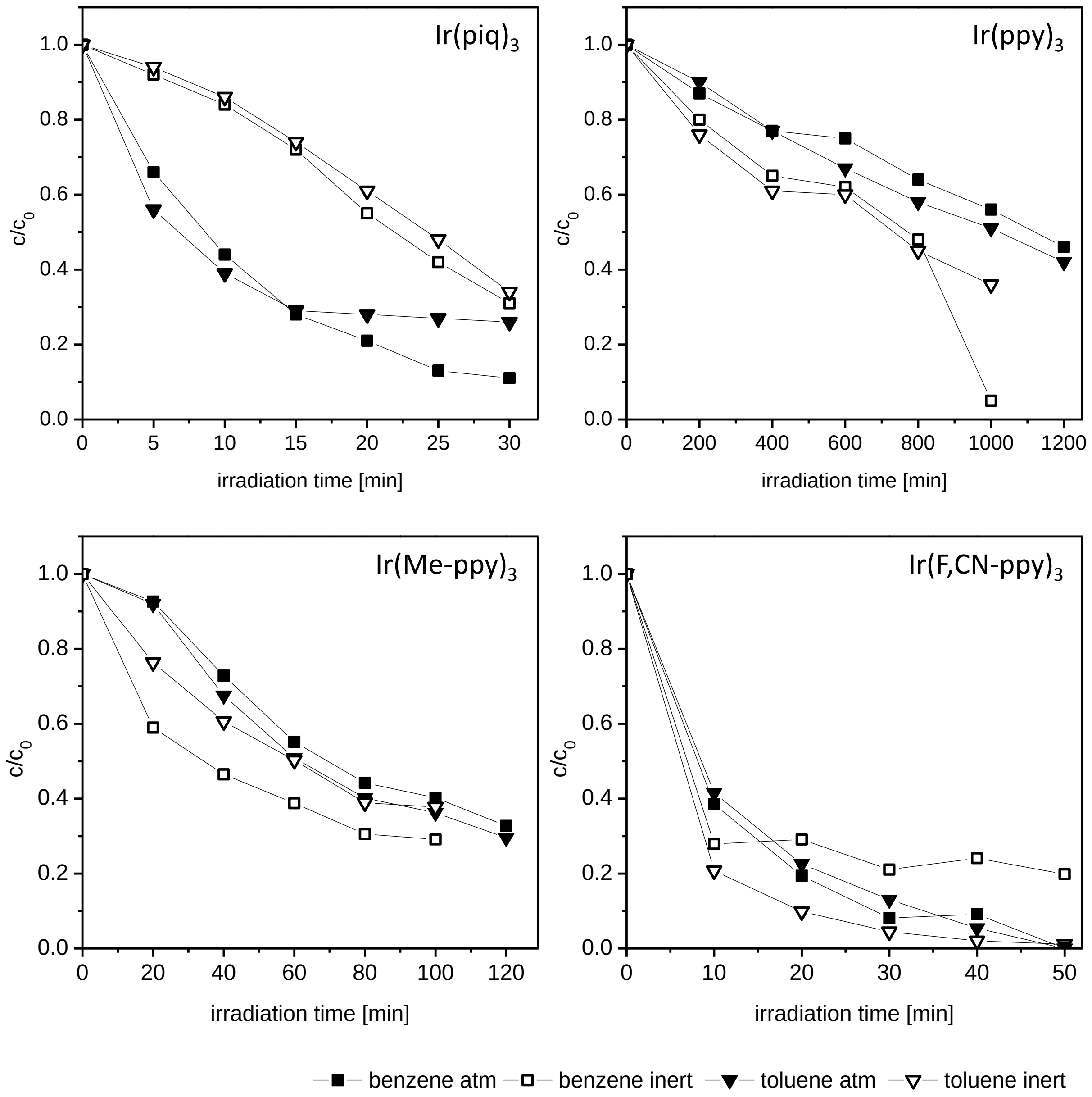

Supplement: File 1 — Reproducibility of degradation studies and degradation curves. [file Beilstein_J_Org_Chem-09-2088-s001.pdf]
